# 4-Chloro-*N*′-(2-hydroxy­benzyl­idene)benzohydrazide monohydrate

**DOI:** 10.1107/S1600536808024471

**Published:** 2008-08-06

**Authors:** Jiu-Fu Lu, Suo-Tian Min, Xiao-Hui Ji, Zhong-Hai Dang

**Affiliations:** a School of Chemistry and Environmental Science, Shaanxi University of Technology, Hanzhong 723000, People’s Republic of China

## Abstract

The asymmetric unit of the title compound, C_14_H_11_ClN_2_O_2_·H_2_O, contains a Schiff base mol­ecule and a water mol­ecule of crystallization. The dihedral angle between the two aromatic rings is 27.3 (4)°. In the crystal structure, mol­ecules are linked into a two-dimensional network parallel to the *bc* plane by inter­molecular O—H⋯O and N—H⋯O hydrogen bonds involving the water mol­ecules.

## Related literature

For general background on Schiff bases derived from condensation of aldehydes with benzohydrazides, see: Fun *et al.* (2008 ▶); Alhadi *et al.* (2008 ▶); Ali *et al.* (2007 ▶); Zou *et al.* (2004 ▶); Shan *et al.* (2008 ▶); Bedia *et al.* (2006 ▶); Terzioglu & Gürsoy (2003 ▶). For related structures, see: Nie (2008 ▶); He (2008 ▶); Shi *et al.* (2007 ▶). For bond-length data, see: Allen *et al.* (1987 ▶).
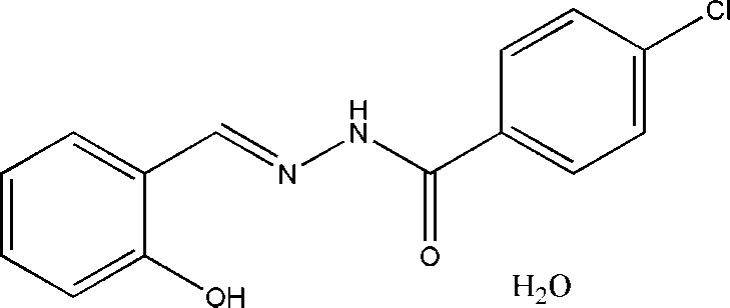


## Experimental

### 

#### Crystal data

C_14_H_11_ClN_2_O_2_·H_2_O
*M* *_r_* = 292.71
Monoclinic, 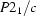

*a* = 22.397 (3) Å
*b* = 4.853 (2) Å
*c* = 12.642 (3) Å
β = 97.15 (3)°
*V* = 1363.4 (7) Å^3^
*Z* = 4
Mo *K*α radiation
μ = 0.29 mm^−1^
*T* = 298 (2) K
0.23 × 0.20 × 0.20 mm

#### Data collection

Bruker APEXII CCD area-detector diffractometer
Absorption correction: multi-scan (*SADABS*; Sheldrick, 2004 ▶) *T*_min_ = 0.937, *T*_max_ = 0.945
10537 measured reflections
2946 independent reflections
1251 reflections with *I* > 2σ(*I*)
*R*_int_ = 0.106

#### Refinement

*R*[*F* ^2^ > 2σ(*F* ^2^)] = 0.068
*wR*(*F* ^2^) = 0.214
*S* = 0.99
2946 reflections
191 parameters
4 restraints
H atoms treated by a mixture of independent and constrained refinement
Δρ_max_ = 0.27 e Å^−3^
Δρ_min_ = −0.27 e Å^−3^

### 

Data collection: *APEX2* (Bruker, 2004 ▶); cell refinement: *SAINT* (Bruker, 2004 ▶); data reduction: *SAINT*; program(s) used to solve structure: *SHELXS97* (Sheldrick, 2008 ▶); program(s) used to refine structure: *SHELXL97* (Sheldrick, 2008 ▶); molecular graphics: *SHELXTL* (Sheldrick, 2008 ▶); software used to prepare material for publication: *SHELXTL*.

## Supplementary Material

Crystal structure: contains datablocks global, I. DOI: 10.1107/S1600536808024471/ci2647sup1.cif

Structure factors: contains datablocks I. DOI: 10.1107/S1600536808024471/ci2647Isup2.hkl

Additional supplementary materials:  crystallographic information; 3D view; checkCIF report

## Figures and Tables

**Table 1:** Hydrogen-bond geometry (Å, °)

*D*—H⋯*A*	*D*—H	H⋯*A*	*D*⋯*A*	*D*—H⋯*A*
O3—H3*A*⋯O2	0.84 (3)	1.97 (3)	2.800 (4)	170 (5)
O3—H3*B*⋯O2^i^	0.85 (3)	2.07 (3)	2.828 (4)	149 (5)
N2—H2⋯O3^ii^	0.90 (1)	1.96 (1)	2.856 (4)	172 (5)
O1—H1⋯N1	0.82	1.96	2.667 (5)	143

Symmetry codes: (i) 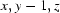; (ii) 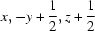.

**Fig. 1.**
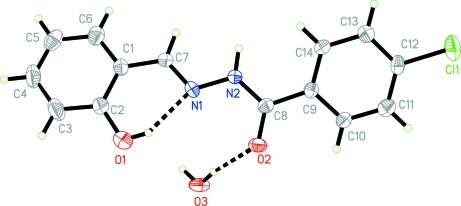
The molecular structure of the title compound, showing 30% probability displacement ellipsoids and the atom-numbering scheme. Dashed lines indicate hydrogen bonds.

**Fig. 2.**
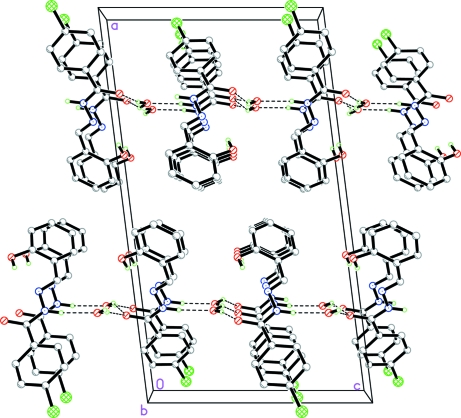
The crystal packing of the title compound, viewed along the b axis. Hydrogen bonds are shown as dashed lines.

## supplementary crystallographic information

### Comment

Schiff bases derived from the condensation of aldehydes with benzohydrazides have been widely investigated, either for their structures (Fun *et al.*, 2008; Alhadi *et al.*,  2008; Ali *et al.*,  2007; Zou *et al.*,  2004; Shan *et al.*,  2008) or for their biological properties (Bedia *et al.*,  2006; Terzioglu & Gürsoy, 2003). This study extends the structural study on such compounds.

The asymmetric unit of the title compound consists of a Schiff base molecule and a water molecule of crystallization (Fig. 1). The bond lengths are within normal values (Allen *et al.*,  1987), and comparable to the values observed in related compounds (Nie, 2008; He, 2008; Shi *et al.*, 2007). The dihedral angle between the two aromatic rings in the Schiff base molecule is 27.3 (4)°. An intramolecular O—H···N hydrogen bond is observed.

In the crystal structure, the molecules are linked into a two-dimensional network parallel to the *bc* plane by intermolecular O–H···O and N–H···O hydrogen bonds (Table 1) involving the water molecules (Fig. 2).

### Experimental

The title compound was prepared by the Schiff base condensation of salicylaldehyde (0.1 mol) and 4-chlorobenzohydrazide (0.1 mmol) in ethanol (50 ml). The excess ethanol was removed by distillation. The colourless solid formed was filtered and washed with ethanol. Single crystals suitable for X-ray diffraction were obtained by slow evaporation of an ethanol solution at room temperature.

### Refinement

The imino and water H atoms were located in a difference map and refined with N–H, O–H and H···H distances restrained to 0.90 (1), 0.85 (1), and 1.37 (2)Å, respectively. The other H atoms were positioned geometrically [C–H = 0.93 Å and O–H = 0.82Å] and refined using a riding model, with U_iso_(H) = 1.2U_eq_(C) and 1.5U_eq_(O1).

### Crystal data

**Table:**

C_14_H_11_ClN_2_O_2_·H_2_O	*F*_000_ = 608
*M**_r_* = 292.71	*D*_x_ = 1.426 Mg m^−^^3^
Monoclinic, *P*2_1_/*c*	Mo *K*α radiation λ = 0.71073 Å
Hall symbol: -P 2ybc	Cell parameters from 600 reflections
*a* = 22.397 (3) Å	θ = 2.6–24.5º
*b* = 4.853 (2) Å	µ = 0.29 mm^−^^1^
*c* = 12.642 (3) Å	*T* = 298 (2) K
β = 97.15 (3)º	Block, colourless
*V* = 1363.4 (7) Å^3^	0.23 × 0.20 × 0.20 mm
*Z* = 4	

### Data collection

**Table:**

Bruker APEXII CCD area-detector diffractometer	2946 independent reflections
Radiation source: fine-focus sealed tube	1251 reflections with *I* > 2σ(*I*)
Monochromator: graphite	*R*_int_ = 0.106
*T* = 298(2) K	θ_max_ = 27.0º
ω scans	θ_min_ = 0.9º
Absorption correction: multi-scan(SADABS; Sheldrick, 2004)	*h* = −28→28
*T*_min_ = 0.937, *T*_max_ = 0.945	*k* = −6→6
10537 measured reflections	*l* = −15→15

### Refinement

**Table:**

Refinement on *F*^2^	Secondary atom site location: difference Fourier map
Least-squares matrix: full	Hydrogen site location: inferred from neighbouring sites
*R*[*F*^2^ > 2σ(*F*^2^)] = 0.068	H atoms treated by a mixture of independent and constrained refinement
*wR*(*F*^2^) = 0.214	*w* = 1/[σ^2^(*F*_o_^2^) + (0.0902*P*)^2^] where *P* = (*F*_o_^2^ + 2*F*_c_^2^)/3
*S* = 0.99	(Δ/σ)_max_ = 0.001
2946 reflections	Δρ_max_ = 0.27 e Å^−^^3^
191 parameters	Δρ_min_ = −0.27 e Å^−^^3^
4 restraints	Extinction correction: none
Primary atom site location: structure-invariant direct methods	

### Special details

**Table:**

Geometry. All esds (except the esd in the dihedral angle between two l.s. planes) are estimated using the full covariance matrix. The cell esds are taken into account individually in the estimation of esds in distances, angles and torsion angles; correlations between esds in cell parameters are only used when they are defined by crystal symmetry. An approximate (isotropic) treatment of cell esds is used for estimating esds involving l.s. planes.
Refinement. Refinement of F^2^ against ALL reflections. The weighted R-factor wR and goodness of fit S are based on F^2^, conventional R-factors R are based on F, with F set to zero for negative F^2^. The threshold expression of F^2^ > 2sigma(F^2^) is used only for calculating R-factors(gt) etc. and is not relevant to the choice of reflections for refinement. R-factors based on F^2^ are statistically about twice as large as those based on F, and R- factors based on ALL data will be even larger.

### Fractional atomic coordinates and isotropic or equivalent isotropic displacement parameters (Å^2^)

**Table:**

	*x*	*y*	*z*	*U*_iso_*/*U*_eq_	
Cl1	0.02717 (6)	1.4002 (3)	0.66329 (12)	0.0731 (5)	
N2	0.24096 (15)	0.4463 (8)	0.6210 (3)	0.0392 (9)	
C8	0.21035 (19)	0.5999 (9)	0.5457 (3)	0.0412 (11)	
N1	0.28603 (15)	0.2706 (7)	0.5952 (3)	0.0420 (10)	
O2	0.21956 (14)	0.5852 (6)	0.4508 (2)	0.0538 (9)	
C9	0.16460 (18)	0.7918 (8)	0.5783 (3)	0.0365 (10)	
C14	0.16219 (18)	0.8667 (9)	0.6822 (3)	0.0426 (12)	
H14	0.1893	0.7895	0.7359	0.051*	
C1	0.35917 (19)	−0.0643 (9)	0.6600 (3)	0.0415 (11)	
C7	0.31165 (18)	0.1242 (9)	0.6720 (3)	0.0428 (12)	
H7	0.2987	0.1416	0.7388	0.051*	
O1	0.36902 (17)	0.0745 (9)	0.4810 (3)	0.0779 (12)	
H1	0.3447	0.1897	0.4963	0.117*	
C11	0.0808 (2)	1.0889 (10)	0.5252 (4)	0.0537 (13)	
H11	0.0531	1.1631	0.4720	0.064*	
C12	0.07965 (19)	1.1639 (9)	0.6292 (4)	0.0457 (12)	
C13	0.1200 (2)	1.0561 (10)	0.7088 (4)	0.0512 (13)	
H13	0.1191	1.1086	0.7793	0.061*	
C2	0.3871 (2)	−0.0802 (11)	0.5669 (4)	0.0536 (13)	
C10	0.12292 (19)	0.9047 (10)	0.4998 (3)	0.0466 (12)	
H10	0.1236	0.8543	0.4290	0.056*	
C6	0.3802 (2)	−0.2325 (10)	0.7435 (4)	0.0577 (14)	
H6	0.3624	−0.2236	0.8060	0.069*	
C4	0.4539 (2)	−0.4315 (13)	0.6486 (5)	0.0726 (17)	
H4	0.4851	−0.5558	0.6443	0.087*	
C5	0.4271 (2)	−0.4144 (11)	0.7380 (5)	0.0656 (15)	
H5	0.4402	−0.5255	0.7962	0.079*	
C3	0.4344 (2)	−0.2616 (12)	0.5632 (5)	0.0739 (17)	
H3	0.4536	−0.2697	0.5021	0.089*	
O3	0.23525 (17)	0.0885 (7)	0.3450 (2)	0.0568 (9)	
H2	0.236 (2)	0.445 (11)	0.6905 (12)	0.080*	
H3B	0.245 (2)	−0.049 (5)	0.385 (3)	0.080*	
H3A	0.235 (2)	0.234 (5)	0.381 (3)	0.080*	

### Atomic displacement parameters (Å^2^)

**Table:**

	*U*^11^	*U*^22^	*U*^33^	*U*^12^	*U*^13^	*U*^23^
Cl1	0.0598 (8)	0.0587 (9)	0.1059 (12)	0.0176 (7)	0.0301 (8)	0.0051 (9)
N2	0.042 (2)	0.044 (2)	0.0329 (19)	0.0072 (18)	0.0065 (17)	0.002 (2)
C8	0.044 (3)	0.040 (3)	0.039 (3)	0.000 (2)	0.003 (2)	0.004 (2)
N1	0.041 (2)	0.042 (2)	0.044 (2)	0.0057 (19)	0.0121 (18)	−0.006 (2)
O2	0.078 (2)	0.050 (2)	0.0345 (18)	0.0109 (18)	0.0142 (16)	0.0034 (16)
C9	0.038 (2)	0.030 (3)	0.041 (3)	−0.002 (2)	0.005 (2)	0.003 (2)
C14	0.045 (3)	0.046 (3)	0.035 (3)	0.005 (2)	−0.001 (2)	0.004 (2)
C1	0.037 (2)	0.038 (3)	0.049 (3)	−0.001 (2)	0.003 (2)	−0.007 (2)
C7	0.044 (3)	0.052 (3)	0.033 (2)	0.005 (2)	0.005 (2)	−0.001 (2)
O1	0.084 (3)	0.098 (3)	0.054 (2)	0.030 (2)	0.0195 (19)	0.002 (2)
C11	0.050 (3)	0.058 (3)	0.053 (3)	0.001 (3)	0.001 (2)	0.005 (3)
C12	0.040 (2)	0.033 (3)	0.065 (3)	0.004 (2)	0.010 (2)	0.007 (2)
C13	0.053 (3)	0.051 (3)	0.050 (3)	0.006 (3)	0.010 (2)	−0.001 (3)
C2	0.051 (3)	0.061 (4)	0.048 (3)	0.009 (3)	0.004 (2)	−0.005 (3)
C10	0.044 (3)	0.054 (3)	0.041 (3)	0.007 (3)	0.003 (2)	0.004 (3)
C6	0.050 (3)	0.055 (3)	0.066 (3)	0.003 (3)	−0.002 (3)	−0.005 (3)
C4	0.051 (3)	0.068 (4)	0.096 (5)	0.019 (3)	−0.001 (3)	−0.024 (4)
C5	0.063 (4)	0.050 (4)	0.077 (4)	−0.001 (3)	−0.019 (3)	−0.002 (3)
C3	0.064 (4)	0.078 (4)	0.083 (4)	0.017 (3)	0.020 (3)	−0.025 (4)
O3	0.091 (2)	0.047 (2)	0.0328 (17)	−0.007 (2)	0.0107 (17)	−0.0014 (17)

### Geometric parameters (Å, °)

**Table:**

Cl1—C12	1.735 (4)	C11—C10	1.366 (6)
N2—C8	1.330 (5)	C11—C12	1.367 (6)
N2—N1	1.391 (5)	C11—H11	0.93
N2—H2	0.899 (10)	C12—C13	1.370 (6)
C8—O2	1.245 (5)	C13—H13	0.93
C8—C9	1.481 (6)	C2—C3	1.384 (7)
N1—C7	1.280 (5)	C10—H10	0.93
C9—C14	1.370 (6)	C6—C5	1.380 (7)
C9—C10	1.388 (6)	C6—H6	0.93
C14—C13	1.390 (6)	C4—C5	1.346 (7)
C14—H14	0.93	C4—C3	1.386 (7)
C1—C6	1.371 (6)	C4—H4	0.93
C1—C2	1.401 (6)	C5—H5	0.93
C1—C7	1.426 (6)	C3—H3	0.93
C7—H7	0.93	O3—H3B	0.85 (3)
O1—C2	1.340 (5)	O3—H3A	0.84 (3)
O1—H1	0.82		
			
C8—N2—N1	120.0 (3)	C11—C12—Cl1	120.7 (4)
C8—N2—H2	126 (3)	C13—C12—Cl1	118.3 (4)
N1—N2—H2	114 (3)	C12—C13—C14	118.8 (4)
O2—C8—N2	121.7 (4)	C12—C13—H13	120.6
O2—C8—C9	120.5 (4)	C14—C13—H13	120.6
N2—C8—C9	117.8 (4)	O1—C2—C3	119.0 (5)
C7—N1—N2	115.6 (3)	O1—C2—C1	121.9 (4)
C14—C9—C10	118.5 (4)	C3—C2—C1	119.1 (5)
C14—C9—C8	123.0 (4)	C11—C10—C9	120.8 (4)
C10—C9—C8	118.4 (4)	C11—C10—H10	119.6
C9—C14—C13	121.1 (4)	C9—C10—H10	119.6
C9—C14—H14	119.5	C1—C6—C5	122.2 (5)
C13—C14—H14	119.5	C1—C6—H6	118.9
C6—C1—C2	117.9 (4)	C5—C6—H6	118.9
C6—C1—C7	119.3 (4)	C5—C4—C3	119.1 (5)
C2—C1—C7	122.8 (4)	C5—C4—H4	120.4
N1—C7—C1	123.0 (4)	C3—C4—H4	120.4
N1—C7—H7	118.5	C4—C5—C6	120.3 (5)
C1—C7—H7	118.5	C4—C5—H5	119.9
C2—O1—H1	109.5	C6—C5—H5	119.9
C10—C11—C12	119.8 (4)	C2—C3—C4	121.4 (5)
C10—C11—H11	120.1	C2—C3—H3	119.3
C12—C11—H11	120.1	C4—C3—H3	119.3
C11—C12—C13	121.0 (4)	H3B—O3—H3A	111 (2)

### Hydrogen-bond geometry (Å, °)

**Table:**

*D*—H···*A*	*D*—H	H···*A*	*D*···*A*	*D*—H···*A*
O3—H3A···O2	0.84 (3)	1.97 (3)	2.800 (4)	170 (5)
O3—H3B···O2^i^	0.85 (3)	2.07 (3)	2.828 (4)	149 (5)
N2—H2···O3^ii^	0.90 (1)	1.96 (1)	2.856 (4)	172 (5)
O1—H1···N1	0.82	1.96	2.667 (5)	143

Symmetry codes: (i) *x*, *y*−1, *z*; (ii) *x*, −*y*+1/2, *z*+1/2.
